# ChromoMapper: a new tool to quickly compare large genome assemblies

**DOI:** 10.1093/bioadv/vbag005

**Published:** 2026-01-09

**Authors:** Elvira Toscano, Elena Cimmino, Angelo Boccia, Leandra Sepe, Giovanni Paolella

**Affiliations:** CEINGE—Biotecnologie Avanzate “Franco Salvatore”, Napoli 80145, Italy; Dipartimento di Medicina Molecolare e Biotecnologie Mediche, Università Degli Studi di Napoli Federico II, Napoli 80131, Italy; CEINGE—Biotecnologie Avanzate “Franco Salvatore”, Napoli 80145, Italy; Dipartimento di Medicina Molecolare e Biotecnologie Mediche, Università Degli Studi di Napoli Federico II, Napoli 80131, Italy; CEINGE—Biotecnologie Avanzate “Franco Salvatore”, Napoli 80145, Italy; Dipartimento di Medicina Molecolare e Biotecnologie Mediche, Università Degli Studi di Napoli Federico II, Napoli 80131, Italy; CEINGE—Biotecnologie Avanzate “Franco Salvatore”, Napoli 80145, Italy; Dipartimento di Medicina Molecolare e Biotecnologie Mediche, Università Degli Studi di Napoli Federico II, Napoli 80131, Italy

## Abstract

**Motivation:**

Quality assessment and assembly comparison are essential steps while assembling new genomes. Many tools for evaluating assemblies typically provide synthetic parameters representing assembly quality or overall features, while others provide long detailed files where it is not always easy to identify and visualize the regions of correspondence and difference among different chromosome assemblies.

**Results:**

Here we present *ChromoMapper*, a new tool which scans the output from *QUAST*, as well as other similar alignment description files, to quickly identify and display similarities and differences between the compared assemblies. It uses the information provided about aligned blocks, combined with additional annotations, to represent the main alignment regions at chromosomal or sub-chromosomal scale, highlighting similarities and collinearity between compared sequences, points of inconsistency, discontinuities, repeated regions and interruptions in the assembled sequences.

**Availability and implementation:**

*ChromoMapper* is available at https://chromomapper.ceinge.unina.it/ and via Zenodo (https://doi.org/10.5281/zenodo.16778863).

## Introduction

Eukaryotic genome assembly procedures strongly depend on quality assessment and assembly evaluation steps (Kim and Kim 2022, Jung *et al.* 2020, Zhang *et al.* 2023). Commonly used tools include reference-free ones, which map reads onto the final assembly to check consistency and detect errors (Hunt *et al.* 2013, Rhie *et al.* 2020, Chen *et al.* 2021, Li *et al.* 2023, Rahman and Pachter 2013, Clark *et al.* 2013) or estimate the completeness of an assembly by looking for conserved genes (Parra *et al.* 2007, Simão *et al.* 2015, Parks *et al.* 2015, Seppey *et al.* 2019) or for repeated genomic regions (Ou *et al.* 2018). Other tools use a reference-based approach to compare one or more assemblies with a reference genome; many tools use *nucmer*, an early genome sequence aligner included in *MUMmer* package (Kurtz *et al.* 2004, Marçais *et al.* 2018), or the more recent *minimap2* (Li 2018) to produce raw files containing a list of alignment blocks. The *MUMmer* package has for a long time provided global comparison parameters as well as graphic representations of the aligned genomes directly or through extensions (Kurtz *et al.* 2004). *GAGE*, a study designed to compare the results of different assemblers, provided a set of scripts which support the calculation of a number of metrics, including different types of misassembly errors (Salzberg *et al.* 2012). Some of these methods and quality metrics were later included in *QUAST*, a tool originally designed for bacterial and small eukaryotic assemblies and later upgraded to support large genomic sequences (Gurevich *et al.* 2013, Mikheenko *et al.* 2018). *QUAST* is currently a quite popular and effective tool, able to compare more than one assembly with a user-provided reference genome and to evaluate a good range of metrics both reference-based and reference-free. Some of this information is made available in graphic format taking advantage of Icarus (Mikheenko, Valin, *et al.* 2016), a browser for assembly exploration and evaluation used within *QUAST* to complement its numerical output with graphic visualization of aligned blocks. In the years, *QUAST* generated some “specialized” quality assessment tools: *MetaQUAST* (Mikheenko, Saveliev, *et al.* 2016) to address metagenomic assembly evaluation; *rnaQUAST* for evaluating RNA-Seq assembly quality (Bushmanova *et al.* 2016); *TandemQUAST* which combines evidence from both k-mers and long reads to better identify certain classes of structural misassembly (Mikheenko *et al.* 2020); *WebQUAST*, a web server which allows to remotely execute *QUAST* runs through a graphical interface (Mikheenko *et al.* 2023).

Here we present *ChromoMapper*, a new tool which complements *QUAST* as well as other tools using the *nucmer* alignment file format by quickly identifying major regions of similarity and the main differences between assemblies. It directly reads output files produced by these tools and quickly shows how similarity is distributed along the chromosomes at different levels of detail. The program produces synthetic as well as detailed reports, which provide global genome coverage and chromosome-by-chromosome analyses. Alignment data is provided in tables or as dynamic graphical representations which allow multiscale browsing of the alignment, highlighting the relationship between chromosomes, contigs and alignment blocks. *ChromoMapper* is available as open-source at https://chromomapper.ceinge.unina.it/ and via Zenodo (10.5281/zenodo.16778863).

## Methods

### Software and languages

Most *ChromoMapper* code was written in PHP programming language, taking advantage of its C-like syntax combined to rapid execution and testing. The code uses an object-oriented approach to develop a modular program. In its present form, *ChromoMapper* runs without additional libraries on Unix, macOS, and Windows operating systems. It was developed on PHP 8.2, but it should be compatible with all versions starting from PHP 8.0.

*ChromoMapper* is built by extending *unixCmd*, a PHP class previously developed in our laboratory to build unix command tools and able to receive options and arguments and to execute the requested commands. In addition, it uses library functions and objects developed in the laboratory, in order to manage data and build tables, produce plots and graphical representations and build simple HTML code for creating output pages, as also previously described (Toscano *et al.* 2022, Toscano, Cimmino, *et al.* 2025).

Plots take advantage of the plotly library and its ability in managing dynamic plots, remaining fast even in presence of large amounts of data (Jing *et al.* 2019, Toscano, Cimmino, *et al.* 2025).

### Tool development

*ChromoMapper* includes a number of procedures for processing alignment data and displaying the results in tables containing synthetic or detailed reports as well as in plots.

During the import phase, alignment block data are extracted from tsv files produced by tools, such as *QUAST* or *nucmer* (Kurtz *et al.* 2004, Gurevich *et al.* 2013, Mikheenko *et al.* 2018, Marçais *et al.* 2018) and re-organized according to the chromosome on which they map. In this phase, additional information is added, to provide alignment run data, including:

- experiment date and name;
- name of the reference genome and of mapped assembly;
- path to the alignment file or its number within a multi-alignment run;
- path to *report*, a summary file produced during *QUAST* run;
- path to a *conversion file*, a tsv file defining a list of chromosomes with their lengths.

If not provided in the *conversion* file, the chromosome list is obtained by choosing the largest sequences in the reference assembly, if the number of chromosomes is specified. Alternatively, the import module tries to guess the sequences corresponding to chromosomes in the reference assembly, by sorting the sequences by descending size and assuming that only sequences larger than 50% of the previous one are chromosomes.

For each alignment block, a set of additional parameters are calculated and organized in three groups: block features, block-end annotations and related blocks (Table 1, available as supplementary material at *Bioinformatics Advances* online). Briefly, the block is evaluated and marked in special cases, i.e. if it has a reversed orientation with respect to reference chromosome (*rev*) or if it is the first or last contig block (*ctgSt* and *ctgEnd*). New start and end position in contig coordinates are calculated and added (*S3* and *E3*) according to 5’-3’ reference direction. Block length is calculated in terms of length on contig (*Length*), length on reference (*LenOnChr*), and difference and ratio between block lengths on contigs and reference (*LenDiff* and *LenExcess*). Block comment provided by tools like *QUAST* is read and analysed to extract block end annotations indicating whether the contig block ends because of a translocation (*Transloc*), and indel (*Indel*) or a local misassembly (*LocMis*). Finally, the number of related blocks is analysed by calculating six additional parameters: *n_unrelated* (number of unrelated blocks, i.e. those which map on the same reference contig/scaffold but on different positions), *n_same* (number of blocks mapping exactly on the same reference position), *n_alternatives* (number of blocks completely contained within the analysed block, i.e. those mapping in positions contained in the alignment length of the test block), *n_larger* (number of larger blocks which contain the analysed block, i.e. block whose mapping length on the reference contains the whole length of test block alignment), *n_ovLeft* (number of blocks whose alignment overlaps on the left with the alignment of the test block), *n_ovRight* (number of blocks whose alignment overlaps on the right with the alignment of the test block). The blocks are then typically filtered by selecting those above a defined *Length* (the default size is 10 000 bases) and with *n_larger* equal to zero, before using them for downstream analyses.

**Table 1:** Block statistics computed by *ChromoMapper*.^a^

Block length	Tot length	Genome fraction	*n* blocks	Blocks unique	Blocks repeated	Avg. length	N50 (kb)	L50	N90 (kb)	L90	*n* contigs	*n* ref.	Blocks per contig (avg.)	Blocks per ref. (avg.)	Identity (avg. %)
>500000	4 290 039	0.02	7	3	4	612 863	620.1	4	501.6	7	7	7	1	1	99.0
>200000	61 800 687	0.26	216	135	81	286 114	281.6	83	209.4	186	114	18	2	12	99.0
>100000	131 364 058	0.55	716	496	220	183 469	192.0	236	114.2	594	195	18	4	40	99.0
>50000	182 581 960	0.76	1435	121	414	127 235	153.1	386	66.5	1120	267	18	5	80	98.9
>20000	215 004 619	0.90	2426	1740	686	88 625	126.0	504	39.7	1680	373	18	7	135	98.8
>10000	225 919 664	0.95	3172	2293	879	71 223	119.8	549	30.5	1960	498	18	6	176	98.8
>5000	230 901 531	0.97	3859	2818	1041	59 835	117.4	570	26.7	2117	634	18	6	214	98.8
>2000	234 818 495	0.98	5111	3781	1330	45 944	115.0	587	23.6	2258	885	18	6	284	98.0
>1000	237 112 084	0.99	6738	5099	1639	35 190	114.0	597	21.7	2349	1149	18	6	355	99.0
>500	238 839 707	1.00	9160	7063	2097	26 074	113.0	604	20.0	2424	1823	20	5	458	99.0
>200	238 839 707	1.00	9160	7063	2097	26 074	113.0	604	20.0	2424	1823	20	5	458	99.0
>100	238 839 707	1.00	9160	7063	2097	26 074	113.0	604	20.0	2424	1823	20	5	458	99.0
>0	238 839 707	1.00	9160	7063	2097	26 074	113.0	604	20.0	2424	1823	20	5	458	99.0

^a^Block statistics for a *Bombus impatiens* assembly (Salzberg *et al.* 2012), compared by QUAST using as a reference the GCA_043295415.1 genome available on *ncbi* website. For each block group, reported parameters are: total bases, genome fraction, number of all, unique and repeated blocks, average block length, N50, L50, N90, L90 values, number of assembly and reference sequences on which blocks mapped, average blocks per contig and per chromosome and identity percentage between assembly and reference.

### Production of alignment files

For *Bombus impatiens* and *Staphylococcus aureus*, assemblies produced by (Salzberg *et al.* 2012) using *SOAPdenovo* (Luo *et al.* 2012) and *MSR-CA* (Zimin *et al.* 2013) assembler, respectively, were used and compared by *QUAST* with the corresponding reference genome available on *ncbi* website (GCA_043295415.1, GCF_000013425.1).

Human assemblies were obtained from *ncbi* website: GRCh37 (GCF_000001405.13), GRCh38.p7 (GCF_000001405.33), released from the Genome Reference Consortium (GRC), were compared by *QUAST* with the current reference genome for *Homo sapiens* GRCh38.p14 (GCF_000001405.40), which, in turn was also mapped against itself; human genome sequence produced by Celera in 2001 (GCA_000002115.2) (Venter *et al.* 2001) and GRCh38.p14 were compared with telomere to telomere human genome assembly (GCF_009914755.1) (Nurk *et al.* 2022, Rhie *et al.* 2023), which, again was also mapped against itself.

Assembly statistics and reference based evaluation was obtained by *QUAST* (Gurevich *et al.* 2013, Mikheenko *et al.* 2018), version 5.0.5, by defining the parameters --*eukaryote*, to indicate that the genome is eukaryotic and not circular, affecting gene finding, conserved orthologs finding and contig alignment; --*large*, to indicate that the genome is larger than 100 Mbp, affecting speed and accuracy, imposing --*min-contig* 3000 --*min-alignment* 500 --*extensive-mis-size* 7000 and allowing to identify misassemblies caused by transposable elements and excluding them from the number of misassemblies, --*conserved-genes-finding*, for enabling search for Universal Single-Copy Orthologs using BUSCO (Simão *et al.* 2015, Seppey *et al.* 2019) by working on eukaryotic gene database; --*no-sv*, to skip structural variation finding as it is yet experimental in the used *QUAST* version; --*features* gene, to provide gene positions in the reference genome.

## Results

*ChromoMapper* is a command line tool which evaluates genome assembly, starting from the results of a mapping procedure, previously carried out using *QUAST* (Gurevich *et al.* 2013, Mikheenko *et al.* 2018) or other mapping tools using the format initially proposed by *nucmer* from the *MUMmer* package (Kurtz *et al.* 2004, Marçais *et al.* 2018). It directly reads the output files of the alignment tool to produce reports organized in tables and plots which together give a global view of the assembled genome, as well as detailed information at the level of individual chromosomes, contigs or alignment blocks.

### *ChromoMapper* reads alignment files to analyse results at alignment block level

**Figure 1:**
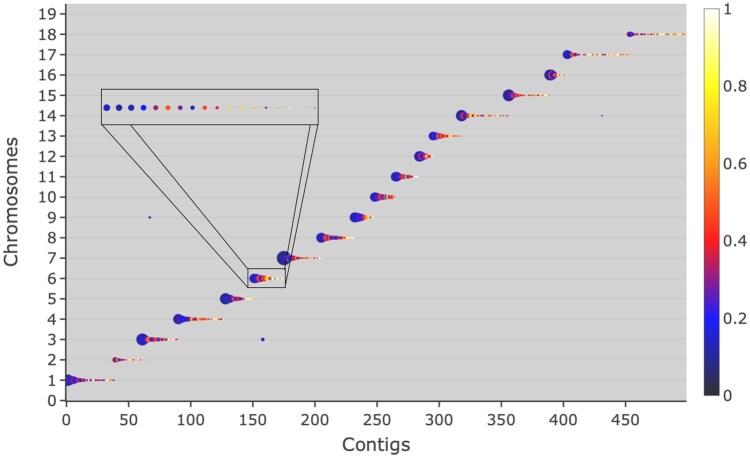
Block level graphical evaluation performed within *ChromoMapper*. For the same experiment as in Table 1, blocks longer than 10 000 bases have been used to plot contigs (x axis) against the reference chromosome (y axis) on which they map, represented as a bubble, whose size depends on the total alignment length and whose colour uses a white-red-blue-black gradient to indicate highly to lowly integrated alignments, where the integrity level is calculated as the ratio between maximum block and contig length. The plot is fully expandable by zooming (see File 1, available as supplementary material at *Bioinformatics Advances* online); the rectangle shows the effect of zooming into the region of chromosome 6.

*ChromoMapper* displays alignment results in tables containing synthetic or detailed reports, as well as in plots. The above-mentioned alignment tools, used to produce the input files, typically provide the results in the form of lists of alignment blocks, i.e. stretches of similarity, ranging in length from tens to millions of bases, which may correspond to entire, or almost entire, contigs/chromosomes but, more often, represent local regions of similarity. Interruptions between blocks occur for a number of reasons, including short insertions or deletions (indels) or bigger inconsistencies, such as large insertions, sequence inversions or jumps to a far-away position on the same chromosome or even a different one. *ChromoMapper* starts from the provided alignment blocks and, for each of them, calculates a set of extra parameters, describing block features, block end annotations and number of related blocks (see Methods). Block statistics are computed organizing blocks in different categories (above or below a length threshold or between two lengths). An example is reported in Table 1, where an assembly from *Bombus impatiens* produced by Salzberg *et al.* (2012) was compared by *QUAST* with a reference genome for this species on *ncbi* website (GCA_043295415.1). Alignment blocks are categorized according to their length and, for each set of blocks larger than a certain length, the following parameters are reported: total length, genome fraction, number of all, unique and repeated blocks, average block length, N50, L50, N90, L90 values, number of assembly and reference sequences on which blocks mapped, average number of blocks for each assembly and reference sequence and identity percentage between the tested assembly and the reference one. Figure 1, available as supplementary material at *Bioinformatics Advances* online, reports a graphical visualization of block features where, for the same experiment as in Table 1, total length (Fig. 1A, available as supplementary material at *Bioinformatics Advances* online) and number of blocks (Fig. 1B, available as supplementary material at *Bioinformatics Advances* online) are calculated by limiting the analysis to blocks longer than a minimum length. The same parameters are reported in Fig. 1C and D, available as supplementary material at *Bioinformatics Advances* online, for blocks whose length is between a lower and an upper limit. The plots show that most of the aligned sequence is in ∼1500 blocks longer than 50 kbases, while less than 20% of it is in smaller blocks, which are of course larger in number (>6000).

**Figure 2:**
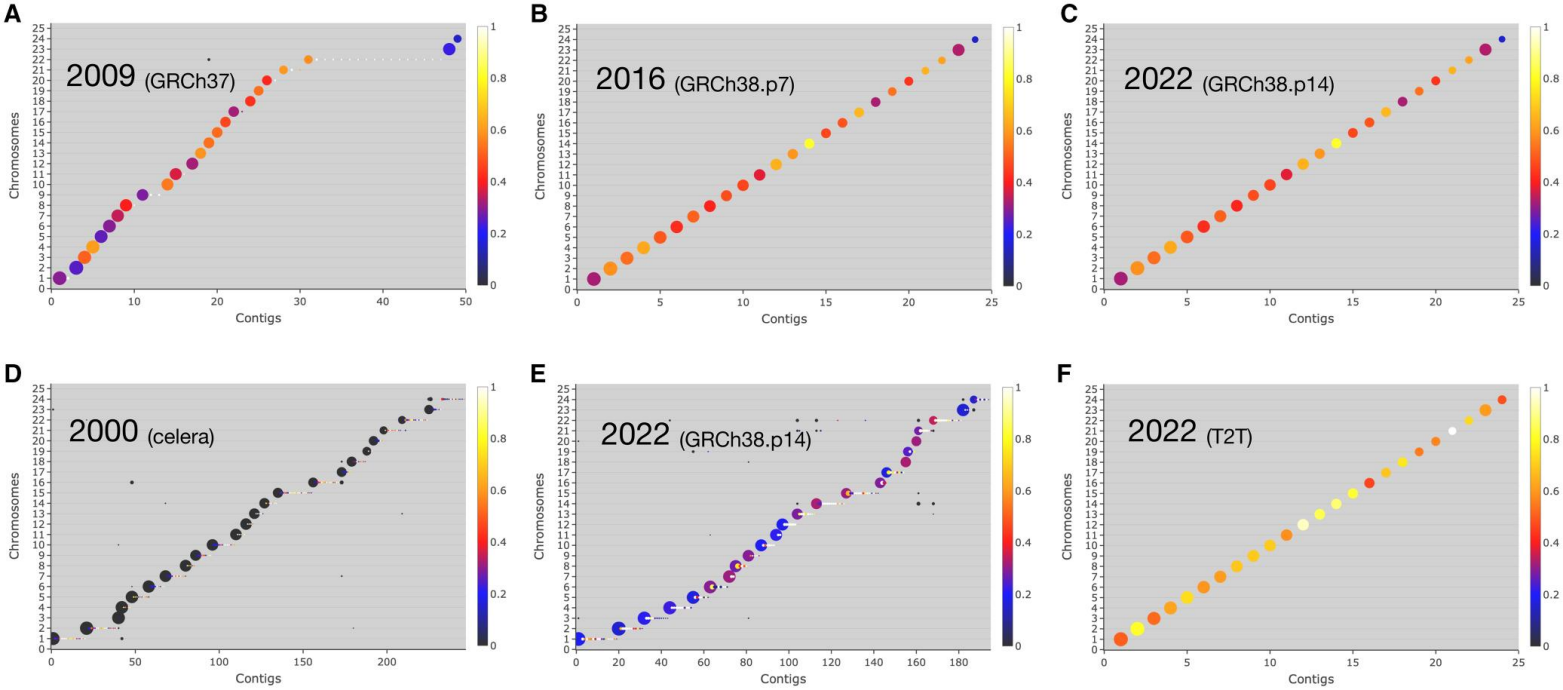
Evaluation of different human genome assemblies. Blocks longer than 10 000 bases have been used to plot contigs (x axis) against the reference chromosome (y axis) on which they map, as in Figure 1. Coordinate 23 and 24 on the y axis correspond to chromosome X and Y, respectively. Assemblies were obtained from ncbi website. (A and B) *Homo sapiens* GRCh37 (GCF_000001405.13) and GRCh38.p7 (GCF_000001405.33) were compared with the current reference genome for GRCh38.p14 (GCF_000001405.40). This last was also mapped against itself (C). (D and E) Genome sequence produced by Celera (GCA_000002115.2) and GRCh38.p14 were compared with telomere-to-telomere (T2T) genome assembly (GCF_009914755.1), which, was also mapped against itself (F).

Figure 1 shows how each block contributes to connect a contig with the chromosome(s) on which it maps, by plotting, at each chromosome/contig intersection, a bubble, whose size depends on the total number of bases, i.e. the sum of the lengths of all blocks contributing to the bubble (File 1, available as supplementary material at *Bioinformatics Advances* online). A multi-colour gradient is used to represent alignment continuity in terms of contig integrity level, calculated as the ratio between length of the largest block and contig length. The gradient goes from white, when a single block is contributing the whole length or nearly, through yellow, red and blue tones, down to black, when a large number of blocks, all 5%–10% of the contig length or smaller, are necessary to produce the full-length alignment. To ease block alignment interpretation, contigs are re-sorted according to the chromosomes they mostly map on: as a consequence, in good alignments, i.e. where there is a high correspondence between the two compared genomes, the bubbles tend to follow the main diagonal, with only a few separated spots corresponding to extra blocks produced when contigs map on more than one chromosome. Blocks are filtered using a minimum size threshold: Fig. 1, where blocks longer than 10 000 bases are used, shows that all chromosomes are covered by a large contig (large bubbles) with integrity level ranging between 0.2 and 0.4. Chromosomes 2 and 18 differ, as they are made by much smaller contigs, whose number importantly increases when the analysis is done on unfiltered blocks (larger than 0) (Fig. 2A, available as supplementary material at *Bioinformatics Advances* online). Increasing the block length threshold up to 20 000, small bubbles disappear while the size of large bubbles is not importantly modified, indicating that the corresponding contigs mainly contain blocks above this length (Fig. 2B, available as supplementary material at *Bioinformatics Advances* online). When blocks longer than 200 000 bases are used, the majority of large bubbles contract or disappear, indicating that the filtered-out alignment blocks contribute an important portion of the alignment length (Fig. 2C, available as supplementary material at *Bioinformatics Advances* online).

This type of plot is also effective at evaluating the alignment of more integrated assemblies where a reduced number of larger contigs/scaffolds are needed to cover the whole length of a reference genome; also in this case, different levels of continuity of an assembly are quickly detected. Figure 2A–C show the analysis of different human genome assemblies, compared by *QUAST* with the current reference genome (GRCh38.p14) released from the Genome Reference Consortium (GRC). The developed representation highlights how the human genome sequence produced in the context of Human Genome Project (Lander *et al.* 2001) evolved over the years and provides a typical example of how *ChromoMapper* may help users resolve assembly comparison scenarios.

GRCh37 assembly covers the reference chromosomes with 50 sequences, with 17 short contigs mapping only on chromosome 22 and with an alignment showing an integrity level ranging between 0.2 and 0.6 (Fig. 2A). GRCh38.p7 assembly is more similar to the current reference assembly (GRCh38.p14), covering its chromosomes with exactly 24 sequences and showing higher integrity levels (0.4–0.8) (Fig. 2B), i.e. longer blocks corresponding to 40%–80% of the largest contigs mapped on the reference chromosomes. These levels of integrity are the same as those observed when GRCh38.p14 sequences are mapped against themselves (Fig. 2C); in both alignments, block interruptions are, in fact, mainly due to the gaps introduced when building the chromosomal scaffolds (see Table 2, available as supplementary material at *Bioinformatics Advances* online, for the 19 blocks on chromosome 1). In Fig. 2E, the same GRCh38.p14 assembly was mapped on the more recent telomere-to-telomere (T2T) assembly (Nurk *et al.* 2022, Rhie *et al.* 2023). As expected, using the new reference, the blocks show lower overall similarity, with the main bubbles similar in size to those of Fig. 2B and C but with integrity levels ranging between 0.20 and 0.35. In addition, blocks from ∼170 additional, much smaller, contigs map on T2T chromosomes, showing that many contigs, not assembled into chromosomes in GRCh38.p14, find correspondence with a chromosome sequence in the T2T assembly. A similar pattern, but much lower in similarity, may be seen when comparing one of the first genome drafts, the one produced by Celera in 2001 (Venter *et al.* 2001), with the same T2T assembly (Fig. 2D). Here, the alignment is highly fragmented, with contigs mapping through blocks consistently smaller than 5% of their length (black bubbles) and over 200 additional very small contigs; Y chromosome coverage is very low. Finally, when T2T assembly is mapped onto itself (Fig. 2F), 24 big bubbles are obtained, as expected. For some chromosomes, the representation shows sequences larger than those from GRCh38.p14: the Y chromosome bubble is visibly larger than in Fig. 2C; a similar result, although less prominent is also visible for chromosomes 1, 9 and 22. Overall, integrity levels are much higher (0.5–1) as, in this case, there are no block interruptions due to scaffold gaps; interestingly, even here, blocks do not span the whole chromosomes, as interruptions still occur in correspondence of centromeric regions, possibly caused by the way the alignment algorithm deals with segmental duplications (Treangen and Salzberg 2011, Phan *et al.* 2015, Rangwala *et al.* 2024).

### Global evaluation of genome assembly

**Figure 3:**
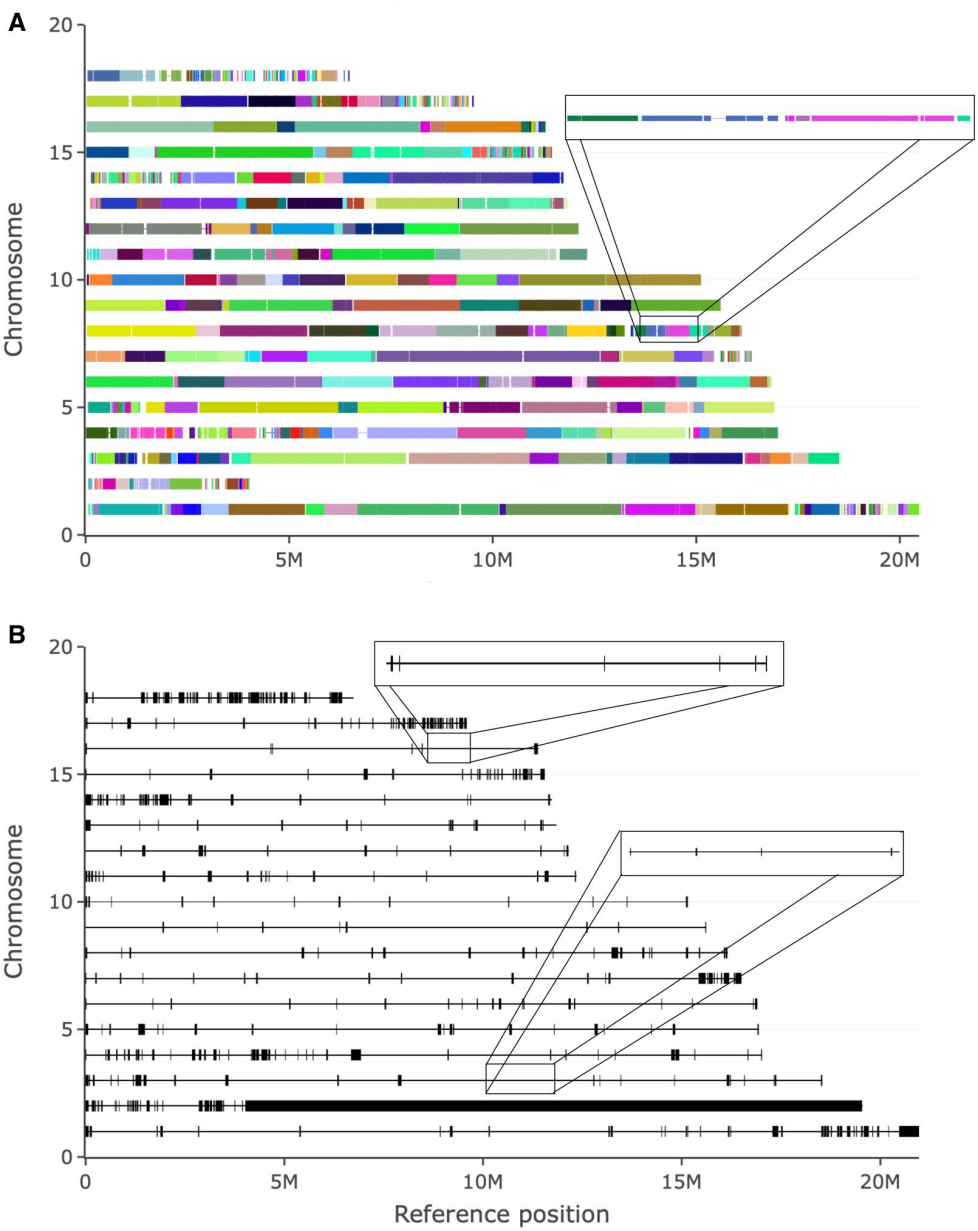
Evaluation of genome aggregation level by ChromoMapper. The same *Bombus impatiens* genome assembly as in Table 1 and Fig. 1. (A) For each chromosome (y axis), alignment blocks are reported as rectangles, coloured according to the contig they belong to and located at the position they map on the reference chromosome (Mbases). Interruptions between contiguous alignment blocks are represented as empty space between rectangles. Horizontal lines connect two contiguous blocks from the same contig. In the zoomed area, a region of chromosome 8 is expanded. (B) For each chromosome reported on the y axis, gaps are represented as black rectangles located at the position they map on the reference chromosome (Mbases). In the rectangles, a zoom on the selected region of chromosome 3 and 16 is reported.

*ChromoMapper* was designed to compare contig or scaffold sets, but also allows to evaluate the integration level of a given assembly by comparing it to a reference genome. A number of different analyses provide a chromosome-by-chromosome summary of reference genome coverage, as well as graphical representations which highlight alignment blocks in detail and the position of gaps in the alignment. In Fig. 3A, for each chromosome (y axis), alignment blocks are reported as rectangles, coloured according to the contig they belong to, while regions of the reference genome not covered by contigs correspond to interruptions between contiguous alignment blocks. Horizontal lines connect two contiguous blocks from the same contig and can be made more visible by zooming in to expand regions of the plot (File 2, available as supplementary material at *Bioinformatics Advances* online). Figure 3B highlights gaps between blocks/contigs, by representing them as black rectangles located at each interruption; also in this case, zooming in allows to see more gaps, typically small enough to be hidden at whole picture level (File 3, available as supplementary material at *Bioinformatics Advances* online). In Fig. 3, an example of such evaluation is reported for the same assembly of the *Bombus impatiens* genome used in Fig. 1 and Table 1. Most chromosomes are covered by 10–20 contigs which together cover essentially their whole length. Chromosome 2 is an exception: it is only covered in the leftmost 4 million bases, while the remaining ∼20 million bases of the reference chromosome correspond to a giant gap in the alignment. These representations also highlight that other chromosomes, such as 1, 4, 7, 14, 17 and 18, present a more fragmented coverage, mainly at their left or right end.

Within *ChromoMapper*, the information about alignment blocks is also summarized in the *Chromosomes* table, which provides a rapid chromosome-by-chromosome view of the major features of the reference genome coverage: leftmost block start (S1) and rightmost block end (E1), fraction of chromosome covered by contigs, number of contigs mapping on each chromosome and their L90 and L50, percent identity between contigs and chromosomes, number of alignment blocks, total aligned block length and reference chromosome length. Such analysis for the *Bombus impatiens* genome (Table 3, available as supplementary material at *Bioinformatics Advances* online) confirms a quite high coverage for most reference chromosomes, with the exception of chromosomes 2 and 18 which show lower coverage values, 14% and 58%, respectively. The number of mapped contigs and the L90 and L50 values reveal a similar level of integration for the different chromosomes, with chromosomes 9, 12 and 16 being much less fragmented than most of the others. The alignment includes 100–200 blocks for most chromosomes.

### Analysis of single chromosomes and contigs

**Figure 4:**
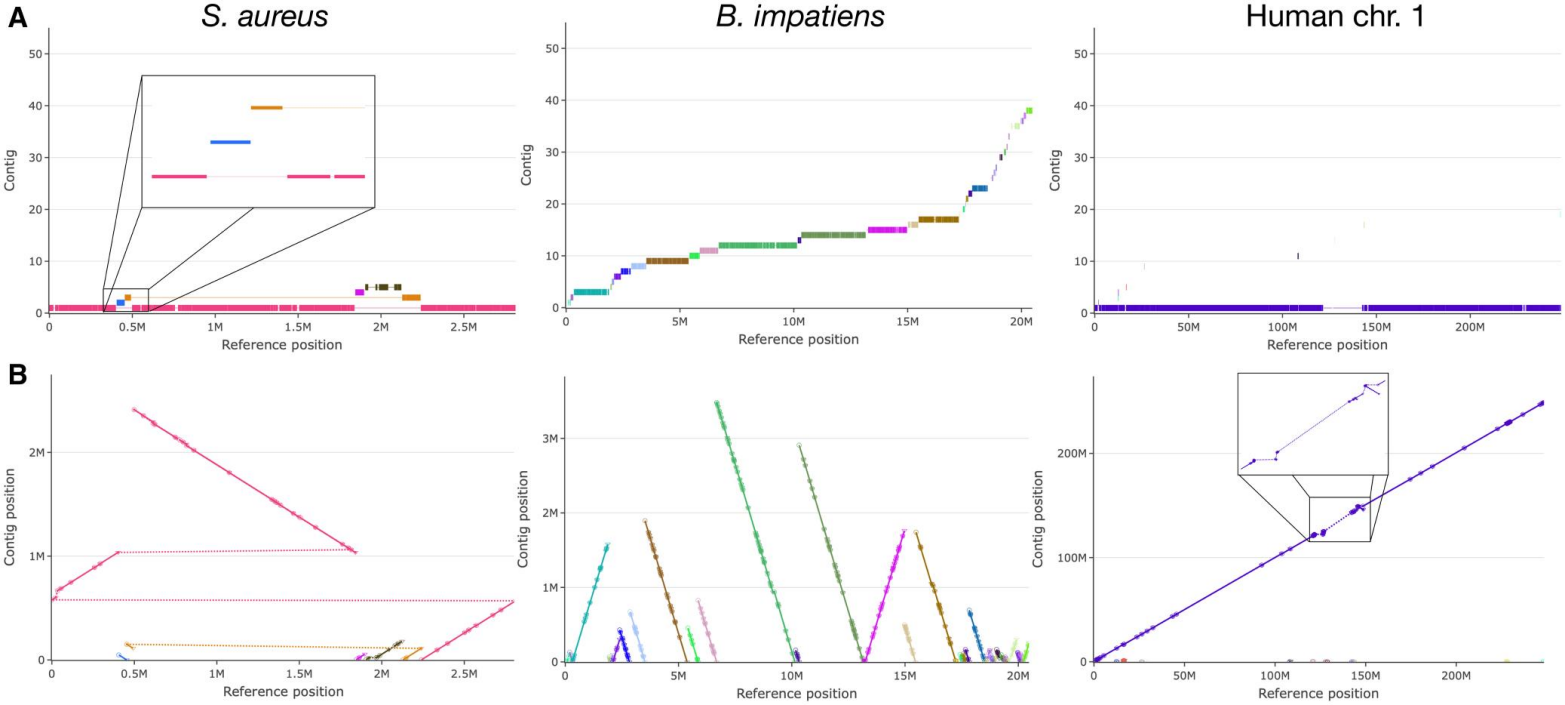
Evaluation of the coverage of single chromosomes in different assemblies. Analysis of *Staphylococcus aureus* genome, chromosome 1 from the *Bombus impatiens* genome assembly, already displayed in Figs. 1 and 3, and human chromosome 1, from GRCh38.p14 compared with telomere-to-telomere (T2T) genome assembly (GCF_009914755.1), is displayed. (A) In each plot, contigs are reported at their y axis level; alignment blocks are reported as coloured rectangles located at reference chromosome position (Mbases) on which they are mapped. Thinner lines are used to connect blocks of the same contigs which are not contiguous on the chromosome. (B) The graph is a dotplot-like representation, where blocks are reported as segments tagged with start (circles) and stop (triangles), located according to contig and reference chromosomes positions (Mbases). Long linear stretches indicate perfect correspondence between contigs and reference genome, insertions and deletions produce jumps from one diagonal line to a parallel one, while inverted blocks are represented as segments with opposite slopes. Dotted lines connect not contiguous blocks on the reference chromosome. In the rectangles, a zoom on the selected regions is reported.

Single chromosome analysis uses two different representations, both shown in Fig. 4. In the first (Fig. 4A), for a given chromosome, each contig is reported at a different y axis coordinate with alignment blocks represented as coloured rectangles located at the reference chromosome position on which they are mapped; thin lines are used to connect blocks of the same contigs when they are not contiguous on the chromosome. The second graph (Fig. 4B) is a dotplot-like representation, where blocks are polarized segments, starting with a circle and ending in a triangle. Long linear stretches indicate perfect correspondence between contigs and reference genome, insertions and deletions produce jumps from one diagonal line to a parallel one, while inverted blocks show opposite slopes. Dotted lines connect adjacent blocks from the same contig when mapped on different parts of the reference chromosome.

Single chromosomes from the same assembly as in Fig. 1 (*Bombus impatiens*) and Fig. 2E (*Homo sapiens*, GRCh38.p14 on T2T) are reported in Fig. 4, together with an assembly for *Staphylococcus aureus* genome, produced by Salzberg *et al.* (2012) and compared by *QUAST* with the corresponding reference genome available on *ncbi* website (GCF_000013425.1). For all examples, the representations allow a quick assessment of the quality of the assembled chromosome sequence. The assembled *Staphylococcus aureus* genome consists of five contigs which map onto the reference sequence, but with a few important differences. The reference genome is mainly covered by a long contig, whose alignment is articulated in four main regions, each composed of several blocks: the first region (∼0.5 Mbases) maps onto the end of the reference sequence (position 2.25–2.8 M); the second is very small and maps onto the initial region of the reference sequence; a small insertion within the contig, represented as a jump to a parallel line, leads to the third alignment region (up to 1 Mbases); finally, the fourth region is the longest one and is represented as a segment with opposite slope, revealing the presence of a large inversion. Regarding *Bombus impatiens* chromosome 1, about 70% of its length is accounted for by less than 20 contigs, with the remaining 30% split among 20 more contigs. Human chromosome 1 from GRCh38.p14 includes one long sequence corresponding to about 90% of the T2T assembly used as reference, interrupted by a large gap between the position 120 to 145 Mbases, corresponding to centromeric satellite repeats and segmental duplications (Nurk *et al.* 2022). Over 10 additional short contigs, from GRCh38.p14 assembly, map onto the same chromosome 1, mainly in its leftmost region. In Fig. 3, available as supplementary material at *Bioinformatics Advances* online, human (GRCh38.p14) chromosome 22 shows a large gap at the leftmost region, corresponding to the first 10 Mbases of the reference (T2T) chromosome sequence. About 5 Mbases are scattered into short blocks belonging to sequences assembled into different chromosomes (13, 14 and 21) in GRCh38.p14. The remaining rightmost region is covered by one long sequence from chromosome 22 of the assembly with a quite continuous alignment, made up from only six blocks. As for chromosome 1, more than 10 additional short contigs mapping onto the chromosome, mainly in its leftmost region, correspond to centromeric satellites and segmental duplications (Nurk *et al.* 2022) (File 4, available as supplementary material at *Bioinformatics Advances* online).

### Tool design and implementation

**Figure 5:**
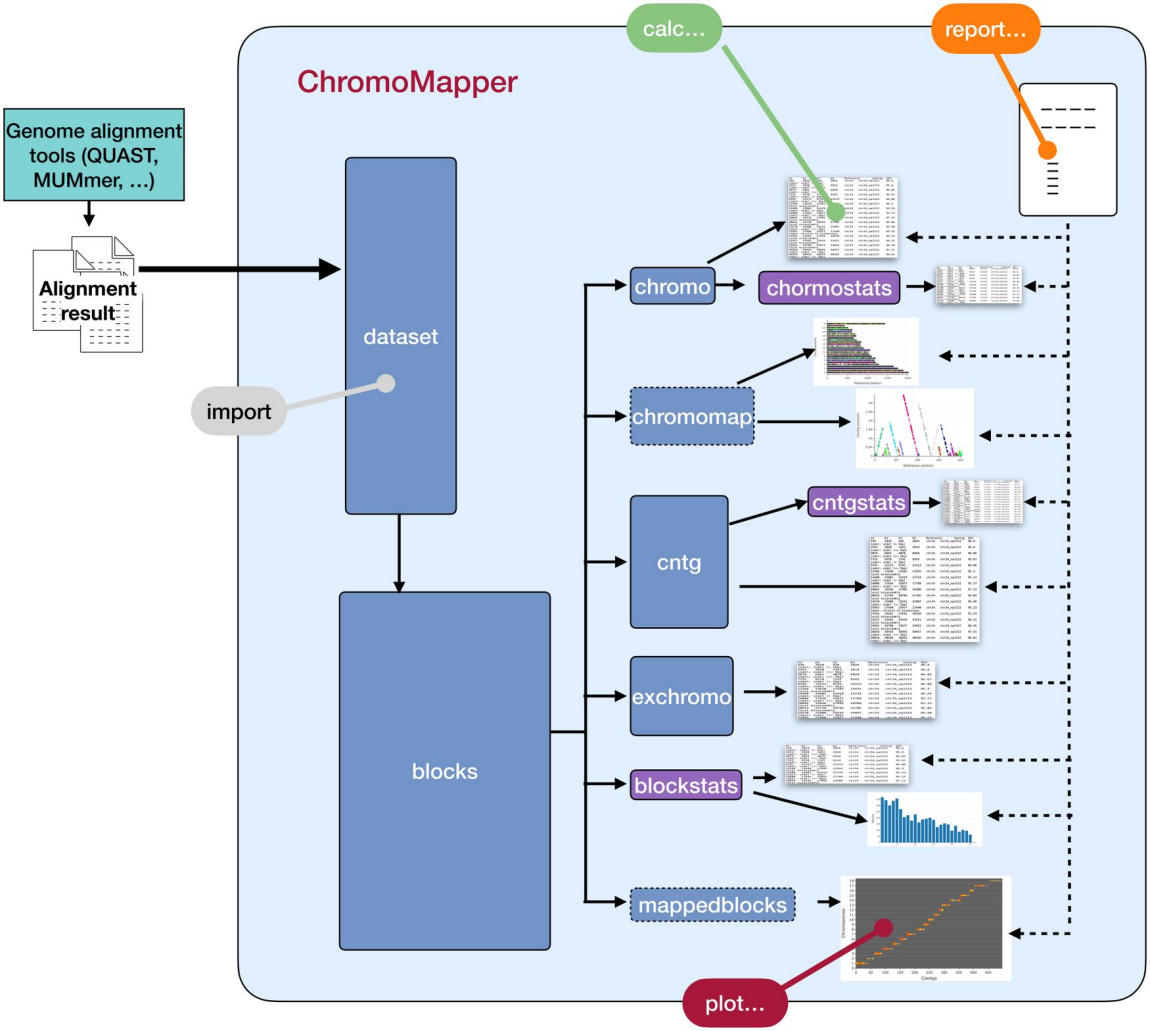
Schematic representation of *ChromoMapper* architecture in terms of commands and data flow. It is based on four principal commands (coloured boxes on the border) which drive the production or the analysis and processing of a dataset. A new alignment run is imported by the *import* command. The *calc* commands produce results as tables. The *plot* commands produce results that are then used to build different types of plots. The *report* commands produce different types of summary reports including multiple tables and plots.

*ChromoMapper* is a command line tool which uses four main commands, *import*, *calc*, *plot* and *report*, for dataset analysis and plot production. A schematic representation of its architecture in terms of commands and data flow is reported in Fig. 5.

The *import* command is used to convert the input data into a pre-processed dataset. A full *QUAST* output directory or a single *QUAST* or *nucmer* alignment file can be directly imported.

The *calc* command produces different results in form of tables, starting from an imported dataset, extended by calculating additional parameters (see Methods). Results include: *blocks*, i.e. the imported alignment blocks; *blockstats*, *cntgstats*, *chromostats*, which compute statistics on aligned blocks, contigs and chromosomes; *cntg*, *chromo* and *exchromo*, which provide a rapid view of the assembly contigs, reference chromosomes or sequences not assembled into chromosomes in the reference genome. This is the result, if a chromosome list was provided as an additional file during the import phase; if not, a chromosome list may still be calculated by guessing it from the sorted reference sequences using a very simple algorithm (see Methods). Options are used to customize the output, provide information about the experiment or filter the dataset by a number of parameters such as chromosome or block length.

The *plot* command uses the available data to calculate the results necessary to produce the various plots, which include: *chromolen*, i.e. a bar plot representing chromosome lengths; *blocksonchrs*, *contigsonchrs* and *gapsonchrs*, which plot alignment blocks, contigs or gaps on all reference chromosomes (Fig. 3); *blocktotlen* and *nblock*, the bar plots used in Fig. 1, available as supplementary material at *Bioinformatics Advances* online, to represent total length and number of blocks; *chromomap* and *contigsonchr* which plot the contigs mapping onto the selected chromosome (Fig. 4); *mappedblocks* which represents the assembly/reference alignment in form of bubble plots, as in Figs. 1 and 2.

Finally, the *report* command produces complete reports in the form of a HTML file containing links to a set of tables and plots. Different report types may be produced by choosing among four different complexity levels.

*ChromoMapper* runs in a PHP (version 8.2) environment without external libraries on Unix, macOS and Windows operating systems and shows very short execution times: on a M2 processor, it typically takes from less than one to a few seconds to produce single plots or tables, up to a few minutes for a full chromosome-by-chromosome report on large mammalian genomes. Execution times and memory usage for different analyses on genome assemblies of different size and quality are reported in Table 4, available as supplementary material at *Bioinformatics Advances* online.

## Discussion

Quality assessment during eukaryotic genome assembly depends on a number of tools which evaluate the resulting genome sequence either directly, using a reference-free approach, or in comparison with reference sequences. Reference-based tools compare one or more assemblies with a reference genome; they typically rely on a fast genomic sequence aligner, such as *nucmer*, included in *MUMmer* system, or *minimap2*, as in the case of *QUAST*, which produce long lists of alignment blocks where it is often not easy to see major regions of similarity or main differences between different assemblies. Within these tools, alignment analysis produces a number of global metrics which readily distinguish low-quality assemblies from better ones, while graphic representation is based on tools such as *Icarus*, tuned to display in great detail each alignment block. However, how contigs are organized, or simply the number and length of the contigs and alignment blocks necessary to cover a given chromosome are often less easy to grasp. Especially when comparing assemblies of similar quality, which typically give similar global scores, differences are often not easy to spot, as it is difficult to immediately realize how similarity is distributed, which regions are better assembled, or simply whether the same contigs and/or alignment blocks are used to build a given chromosome.

The tool described in this work, *ChromoMapper*, uses *QUAST* output files, as well as others in similar format, to quickly identify and display similarities and differences between assemblies. It works on the alignment blocks produced during the alignment phase, analysing the relationship between reference and assembly sequences at different levels: contigs, scaffolds or fully assembled chromosomes are evaluated and displayed considering how they map onto the chromosomes of the reference genome, using user-provided information, such as chromosome name and expected length, or guessing the chromosomes from their size. If the reference sequence is not assembled into chromosomes, *ChromoMapper* can still work on scaffold or contig sets aligned to each other, although, of course, in this case chromosome-related tables cannot be provided.

Within *ChromoMapper*, alignment data are displayed in synthetic form, using tables or plots, where results are organized at different level of detail, going from a global view of the genome down to detailed information about blocks, individual chromosomes or contigs. Chromosome-by-chromosome genome coverage is provided as a summary table, as a whole genome graphic map and as graphical representations of single chromosomes. The global genome plots of Fig. 3 topographically map similarities and differences onto the various chromosomes; the deep zooming level provided by these plots allows to keep all block-level information even within this “globally” oriented representation (Fig. 3A and File 2, available as supplementary material at *Bioinformatics Advances* online). In addition to alignment blocks, spaces between blocks, visualized within the same context, complement the blocks/contig view by stressing the interruptions between contigs (Fig. 3B and File 3, available as supplementary material at *Bioinformatics Advances* online). Graphical representation of single chromosomes generates, for each of them, two different plots, which highlight various alignment features: insertions, deletions, translocations, inversions, etc. (Fig. 4). Also these plots start showing a global chromosome view and may be dynamically expanded by zooming at different levels of resolution, to make even small regions visible and show details not visible at whole picture level (see File 4, available as supplementary material at *Bioinformatics Advances* online).

Overall, the major contribution of *ChromoMapper* is possibly in improving visualization and interpretability of assembly comparison results. The described level of detail is, in fact, better than that provided by a number of tools for visualizing genome alignments, such as the early *MGView*, originally designed to support gap closure of microbial genomes (Herron-Olson *et al.* 2003) or *Synteny Portal* which visualizes synteny blocks by using prebuilt alignments in the *UCSC* genome browser database (Speir *et al.* 2016). More recently, synteny tracks and structural variations among genomes have been also included in full featured genome browsers, like *JBrowse2* (Diesh *et al.* 2023), or comparative genomic viewers, like *CGV* (Rangwala *et al.* 2024), although, this last is only available to display prebuilt whole-genome sequence alignments provided by NCBI rather than newly assembled genomes.

When assembling, an important point deals with alignment blocks which invariably fragment a contig mapping onto a reference sequence: more than the simple number of blocks, their length, level of identity, orientation, distance and the features of the connecting unaligned sequences are indications of alignment quality. Alignment representations provided by *ChromoMapper* (Fig. 4) quickly distinguish long regions of near 100% sequence identity, where blocks are simply indications of point changes, from “difficult” regions where a large number of smaller or much smaller blocks indicate the presence of alternative assembly/mapping as often happens in areas rich in repetitive sequences, such as centromeric regions or X chromosomes. Using a customized bubble plot, *ChromoMapper* can also globally show how each block contributes to connect each contig with the chromosome(s) on which it maps. The examples in Fig. 2 are typical situations where *ChromoMapper* may help users in solving assembly comparison scenarios as they show how this block/contig/chromosome approach quickly highlights similarity, changes and level of integration, when comparing assemblies made at many years distance and with completely different approaches. In addition, even if an assembly is mapped against itself, the developed representation results useful in evaluating the integration level of the chromosome scaffolds by highlighting block interruptions due to gaps introduced when producing scaffolds.

*ChromoMapper* allows to easily monitor the whole assembly process, all the way from contigs to scaffolds to chromosomes. At early stages, mapping assembled contigs onto a reference genome gives a quick view of how much of it is covered, as well as the level of integration, both globally and chromosome by chromosome. Different assemblies may be compared with each other to show alternative assemblies of the same sequences, which is especially useful when no reference sequence is available, or when forcing a *de novo* assembly to potentially highlight structural variations. Assemblies from long-read sequencing, well supported in *ChromoMapper* (Toscano, Sepe, *et al.* 2025), are even more effective as they often tend to be more continuous than those obtained by assembling shorter sequences obtained through other sequencing procedures. When scaffolds or full chromosomes become available, they can quickly be evaluated by taking advantage of tables and plots. *ChromoMapper* usage is very easy: it uses four main commands, *import*, *calc*, *plot* and *report*, for dataset import and analysis and plot production. It integrates all the results in a comprehensive report, containing links to sets of tables and plots, but also allows to produce plots or tables singularly. It is a flexible and effective system to analyse assembly structure and alignment quality by combining statistical evaluation and graphical visualizations, across a wide range of genome sizes (further documentation and usage examples can be found at https://chromomapper.ceinge.unina.it/). The main limitations are directly linked to what is the main focus of the application, i.e. the assessment of the quality of reasonably continuous partial or full genome assemblies, as many tables and visualizations are organized at the chromosome level. In this respect, very discontinuous assemblies of large genomes end up by producing millions, rather than thousands, of blocks resulting, in the worst case, in long processing times and very crowded plots. Even in this case, though, execution time, although slower (minutes or tens of minutes rather than seconds), remains still usable and the zoom features of the plots help getting at the results anyway.

Of course, genome assembly analysis is necessarily defined and possibly limited by the features of the alignment tool used to produce the alignment files, although *QUAST* and other currently available tools are mature alignment programs which can effectively process assembled genomes, even of large size such as mammalian genome. *ChromoMapper* directly supports alignment files produced by the previously mentioned programs which use the file format initially proposed by *nucmer*; however, it should not be too difficult to support other file formats by converting them before importing or by modifying the import routine. An obvious expansion for a visualization tool producing plots is to make it available as an accessible online service, which could interactively provide rapid recalculation and allow multiscale access to the alignment features through the plot-zooming features. Finally, possible further expansions for *ChromoMapper* could be in supporting the analysis of metagenomic assemblies to compare genomes from different pathogens or even in evaluating the different assembly steps from contig assembly, starting from short and/or long reads, up to scaffold/chromosome reconstruction, obtained by using Hi-C strategies or others.

## Supplementary Material

vbag005_Supplementary_Data

## Data Availability

The software and sample data reported in this article are available at https://chromomapper.ceinge.unina.it/ and in *Zenodo* at https://zenodo.org, and can be accessed with *10.5281/zenodo.16778863*.
