# Supplementary material for: ChromoMapper: a new tool to quickly compare large genome assemblies
Source: Bioinform Adv. 2026 Jan 9;6(1):vbag005. doi: 10.1093/bioadv/vbag005 (PMC12947579; doi:10.1093/bioadv/vbag005)
Supplement: vbag005_Supplementary_Data [file vbag005_supplementary_data.zip › SupplementaryMaterial/SupFile1-mappedblocks.html]

x

Size group: above   
Length category: >10000   
Blocks Type: all   
Blocks mapped on: chromosomes   
No zero: yes
