# Supplementary material for: ChromoMapper: a new tool to quickly compare large genome assemblies
Source: Bioinform Adv. 2026 Jan 9;6(1):vbag005. doi: 10.1093/bioadv/vbag005 (PMC12947579; doi:10.1093/bioadv/vbag005)
Supplement: vbag005_Supplementary_Data [file vbag005_supplementary_data.zip › SupplementaryMaterial/SupFile4-chr22.html]

x

Chromosome: 22   
Blocks Min Length: 10000
