# Supplementary material for: ChromoMapper: a new tool to quickly compare large genome assemblies
Source: Bioinform Adv. 2026 Jan 9;6(1):vbag005. doi: 10.1093/bioadv/vbag005 (PMC12947579; doi:10.1093/bioadv/vbag005)
Supplement: vbag005_Supplementary_Data [file vbag005_supplementary_data.zip › SupplementaryMaterial/Supplementary Material.pdf]

Supplementary material

Group	Parameter	Description
block features	rev	indicates whether the contig block has a reversed orientation respect to reference chromosome
	ctgSt	indicates whether the block is the first one of the contig
	ctgEnd	indicates whether the block is the last one of the contig
	S3	block start position in contig coordinates adjusted considering block orientation
	E3	block end position in contig coordinates adjusted considering block orientation
	Length	block length on contig
	LenOnChr	block length on reference
	LenDiff	difference between block lengths on contigs and reference
	LenExcess	ratio between block lengths on contigs and reference
block end annotations	Transloc	indicates whether the contig block ends because of a translocation
	Indel	indicates whether the contig block ends because of a indel
	LocMis	indicates whether the contig block ends because of a local misassembly
related blocks	nUnrel	number of unrelated blocks, i.e. those which map on the same reference contig/ scaffold but on different positions.
	nSame	number of blocks mapping exactly on the same reference position.
	nAlt	number of blocks completely contained within the analysed block, i.e. those mapping in positions contained in the alignment length of the test block.
	nLarger	number of larger blocks which contain the analysed block, i.e. block whose mapping length on the reference contains the whole length of test block alignment.
	nOvLeft	number of blocks whose alignment overlaps on the left with the alignment of the test block.
	nOvRight	number of blocks whose alignment overlaps on the right with the alignment of the test block.

Supplementary Table 1. Block parameters computed within *ChromoMapper*. The set of additional parameters, calculated for each alignment block, is organized in three groups: block features, block end annotations and related blocks.

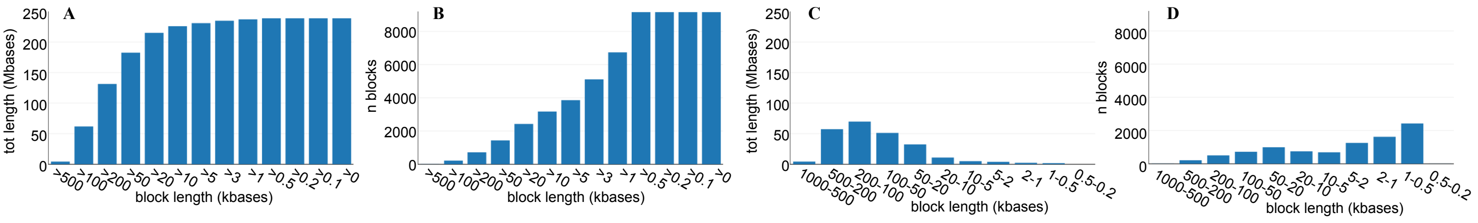

Supplementary Figure 1. Block statistics calculated within *ChromoMapper*. (A-B) Total length (A) and number of blocks (B) were calculated, for *Bombus impatiens* assembly aligned to the reference genome (same alignment as in Table and Figure 1), by limiting the analysis to blocks longer than a threshold. (C and D) The same parameters calculated for blocks of length comprised between a lower and an upper limit.

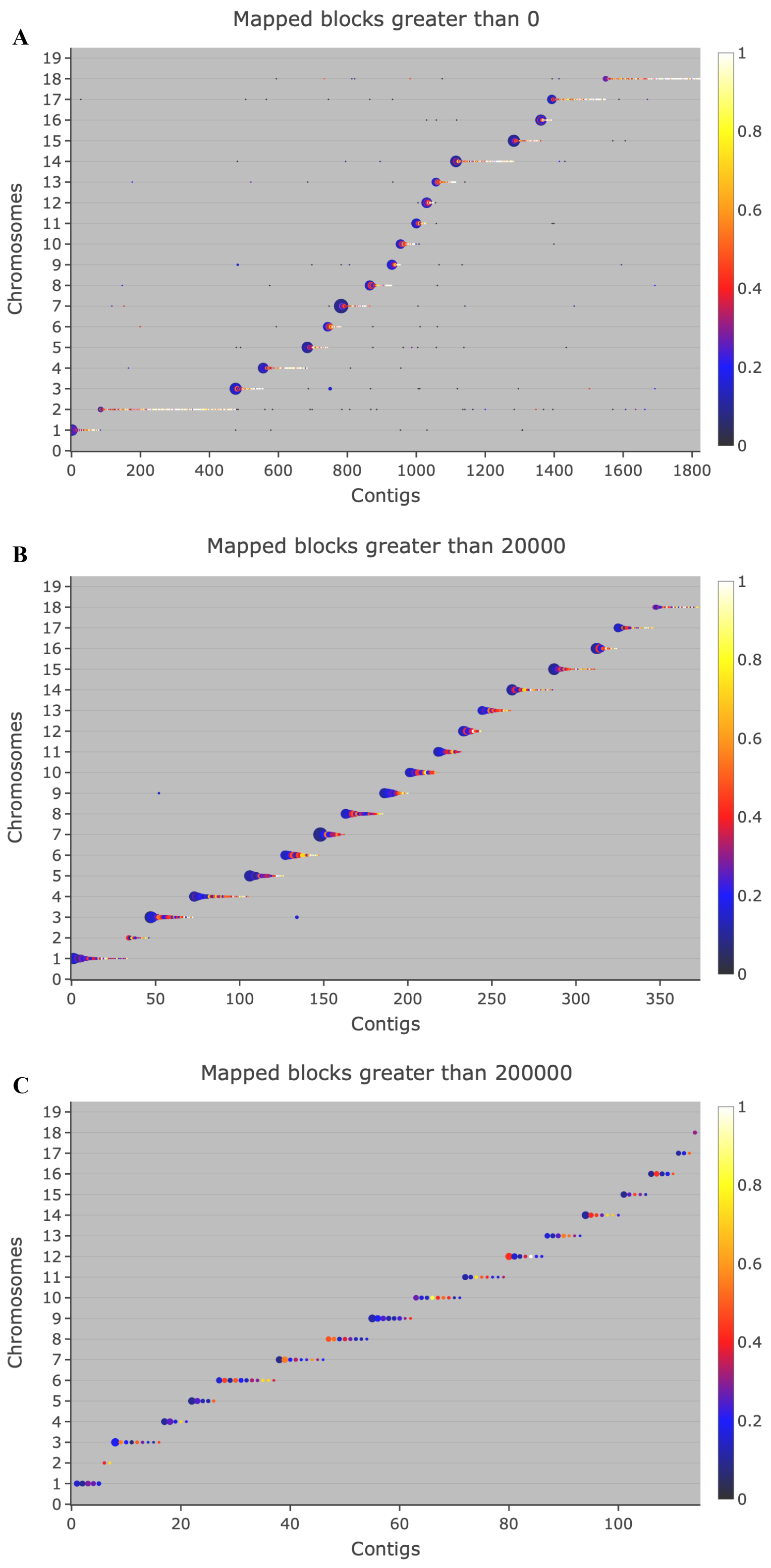

Supplementary Figure 2. Bubble plot for the *Bombus impatiens* assembly by using different block length thresholds. Blocks from *Bombus impatiens* assembly aligned to the reference genome (same alignment as in Table and Figure 1) were used to relate contigs (x axis) with the chromosomes on which they map on the reference genome (y axis). Block are filtered according to different length thresholds: (A) all blocks, (B) blocks larger than 20000 bases and (C) blocks larger than 200000 bases. The alignment blocks from each contig are represented as bubbles, whose size depends on the number of bases involved in the alignment and using a white-red-blue-black gradient to indicate the integrity level, calculated as the ratio between largest block length and contig length.

ID	Reference	refID	ctgID	S1	E1	S2	E2	Length	RDistance	CDistance
1	NC_000001.11	1	1	10001	207666	10001	207666	197666	10000	10000
2	NC_000001.11	1	1	257667	297968	257667	297968	40302	50000	50000
3	NC_000001.11	1	1	347969	535988	347969	535988	188020	50000	50000
4	NC_000001.11	1	1	585989	2702781	585989	2702781	2116793	50000	50000
5	NC_000001.11	1	1	2746291	12954384	2746291	12954384	10208094	43509	43509
6	NC_000001.11	1	1	13004385	16799163	13004385	16799163	3794779	50000	50000
7	NC_000001.11	1	1	16849164	29552233	16849164	29552233	12703070	50000	50000
8	NC_000001.11	1	1	29553836	56365565	29553836	56365565	26811730	1602	1602
9	NC_000001.11	1	1	56365566	121890873	56365566	121890873	65525308	0	0
10	NC_000001.11	1	1	121890976	121916621	121890976	121916621	25646	102	102
11	NC_000001.11	1	1	121916727	121976459	121916727	121976459	59733	105	105
12	NC_000001.11	1	1	122026460	124977944	122026460	124977944	2951485	50000	50000
13	NC_000001.11	1	1	124978327	125013060	124978327	125013060	34734	382	382
14	NC_000001.11	1	1	125013224	125130246	125013224	125130246	117023	163	163
15	NC_000001.11	1	1	125131848	125171347	125131848	125171347	39500	1601	1601
16	NC_000001.11	1	1	125173584	125184587	125173584	125184587	11004	2236	2236
17	NC_000001.11	1	1	143184588	223558935	143184588	223558935	80374348	18000000	18000000
18	NC_000001.11	1	1	223608936	228558364	223608936	228558364	4949429	50000	50000
19	NC_000001.11	1	1	228608365	248946422	228608365	248946422	20338058	50000	50000

Supplementary Table 2. Chromosome 1 blocks for GRCH38.p14 mapped against itself. The 19 alignment blocks on chromosome 1 of the human genome assembly GRCH38.p14 (GCF_000001405.40) mapped against itself are reported, together with additional parameters calculated within *ChromoMapper*: reference name, reference and config ID, block start and end both in reference and contig coordinates (S1, E1, S2, E2), block length and distance between contig start position and end position of the previous alignment block, calculated both on reference (RDistance) and contig (CDistance).

refID	S1	E1	coverage (%)	n contigs	L90	L50	identity (%)	n blocks	length	ref length
Chr1	76,356	20,472,4120	89.95	38	38	5	98.61	284	18,973,032	20,973,032
Chr2	74,718	4,017,465	14.21	22	22	22	98.21	69	2,777,468	19,540,043
Chr3	64,700	18,504,133	94.86	30	19	4	98.95	229	17,584,738	18,537,726
Chr4	16,478	16,998,875	88.66	38	38	6	98.51	239	15,092,788	17,022,635
Chr5	64,478	16,908,150	94.33	23	14	4	98.68	211	15,979,564	16,939,463
Chr6	19,348	16,844,591	96.58	24	12	5	98.70	198	16,324,184	16,902,051
Chr7	15,875	16,350,493	92.97	30	12	3	98.94	198	15,342,642	16,503,469
Chr8	39,390	16,106,265	94.04	27	18	5	98.77	214	15,186,934	16,149,712
Chr9	2,384	15,591,990	98.93	17	9	4	98.98	186	15,447,004	15,614,383
Chr10	37,374	15,106,655	97.50	17	13	5	99.02	178	14,775,347	15,154,412
Chr11	40,419	12,312,007	94.06	19	12	4	99.00	153	11,608,087	12,341,294
Chr12	8,568	12,101,676	96.10	11	8	3	99.07	122	11,676,021	12,150,031
Chr13	122,842	11,837,877	94.70	23	13	4	98.76	140	11,212,643	11,839,581
Chr14	141,885	11,728,329	89.45	39	39	4	99.07	167	10,495,834	11,733,401
Chr15	20,140	11,444,684	90.97	33	27	3	98.81	165	10,508,115	11,551,769
Chr16	30,377	11,288,754	98.38	14	6	2	98.84	132	11,196,008	11,380,321
Chr17	27,229	9,524,998	82.66	50	50	3	98.58	175	7,927,715	9,590,353
Chr18	52,169	6,472,525	58.22	46	46	18	98.41	112	3,928,982	6,731,488

Supplementary Table 3. *Chromosomes* table produced by *ChromoMapper*. Chromosome-by-chromosome view of the major features of the *Bombus impatiens* genome coverage by the same assembly as in Table and Figure 1. For each chromosome, parameters are reported: leftmost block start (S1) and rightmost block end (E1), fraction of chromosome covered by contigs, number of contigs mapping on each chromosome and their L90 and L50, percent identity between contigs and chromosomes, number of alignment blocks, total aligned block length and reference chromosome length.

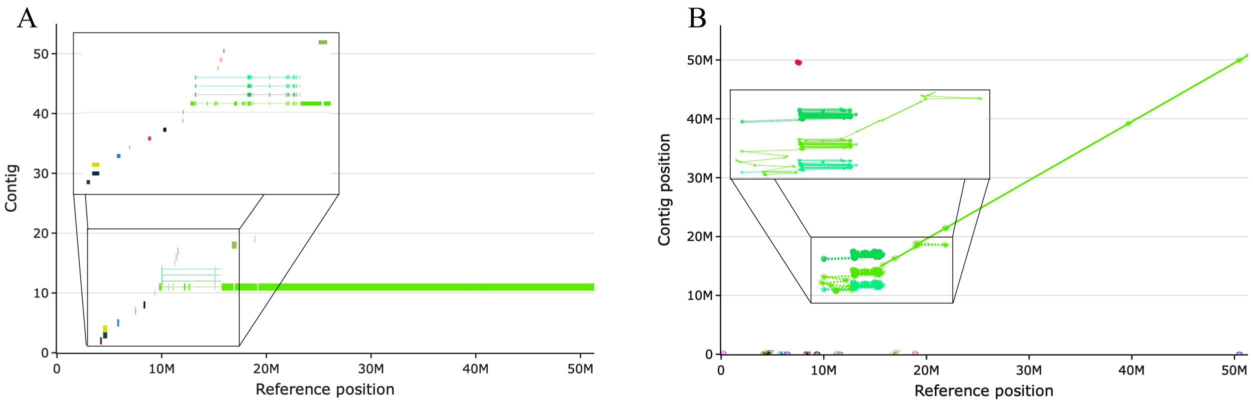

Supplementary Figure 3. Evaluation of the coverage of human chromosome 22. Analysis of human chromosome 22, from GRCh38.p14 compared with telomere-to-telomere (T2T) genome assembly (GCF_009914755.1), is displayed. (A) Contigs are reported at their y axis level; alignment blocks are reported as coloured rectangles located at reference chromosome position (Mbases) on which they are mapped. Thinner lines are used to connect blocks of the same contigs which are not contiguous on the chromosome. (B) The graph is a dotplot-like representation, where blocks are reported as segments tagged with start (circles) and stop (triangles), whose coordinate reported as a function reference chromosome position (Mbases). Long linear stretches indicate perfect correspondence between contigs and reference genome, insertions and deletions produce jumps from one diagonal line to a parallel one, while inverted blocks are represented as segments with opposite slopes. Dotted lines connect not contiguous blocks on the reference chromosome. In the rectangles, a zoom on the selected regions is reported.

Feature	Reference	GCF_000013425.1	GCA_043295415.1	GCF_009914755.1 (T2T)	GCF_000001405.40 (GRCh38.p14)
	Assembly	*S. aureus (MSR-CA)*	*B. impatiens (SOAPdenovo)*	*H. sapiens* - GRCh38.p14	*H. sapiens* - chr 14 (SOAPdenovo)
**Genome size**		2,821,361	266,572,400	3,117,275,501	107,043,718
**Number of ref chromosomes**		1	18	24	1
**Number of contigs**		17	25,528	705	38,477
**Number of blocks**		87	8,793	7,920	13,953
**Importing from local file (time in seconds)**		0.006	0.085	0.12	0.158
**Producing chromo table (time in seconds)**		0.005	0.174	0.213	5.821
**Producing cntg table (time in seconds)**		0.007	0.667	0.959	44.98
**Producing mappedblocks plot (time in seconds)**		0.009	0.581	0.821	39.321
**Producing contigsonchrs plot (time in seconds)**		0.006	0.276	0.265	5.904
**Producing chromomap plot (time in seconds)**		0.006	0.184	0.222	5.767
**Producing report (time in seconds)**		0.026	4.393	6.854	262.481
**Producing table report (time in seconds)**		0.238	4.33	6.492	257.234
**Producing plot report (time in seconds)**		0.036	12.218	17.895	250.937
**Producing full report (time in seconds)**		0.057	15.487	22.23	431.03
**Importing from local file (used memory in MB)**		4.2	35.7	46.1	62.9
**Producing chromo table (used memory in MB)**		4.2	73.4	94.4	119.5
**Producing cntg table (used memory in MB)**		4.2	98.6	134.2	167.8
**Producing mappedblocks plot (used memory in MB)**		4.2	92.3	127.9	169.9
**Producing contigsonchrs plot (used memory in MB)**		4.2	83.9	100.7	127.9
**Producing chromomap plot chr1 (used memory in MB)**		4.2	73.4	94.4	127.9
**Producing report (used memory in MB)**		8.4	159.4	216.0	281.0
**Producing table report (used memory in MB)**		8.4	159.4	216.0	281.0
**Producing plot report (used memory in MB)**		10.5	199.2	262.1	312.5
**Producing full report (used memory in MB)**		14.7	203.4	266.3	316.7

Supplementary Table 4. Execution times and memory usage for different assembly analyses. The table reports the analysis of three assemblies produced by Salzberg et al. (2012) for *S. aureus* and *B. impatiens* and human chromosome 14, assembled by using *MSR-CA* and *SOAPdenovo* assemblers and mapped on the respective reference genomes, GCF_000013425.1, GCA_043295415.1 and GCF_000001405.40. In addition, this last was evaluated and mapped on the T2T human genome assembly (GCF_009914755.1). For each mapping, execution times and memory usage, obtained on a M2 Max Mac Studio, are reported for a number of functions and tasks: import, calculation of *chromo* and *cntg* tables, production of *mappedblocks*, *contigsonchrs* and *chromomap* plots and the building of standard, table, plot and full reports.

Supplementary downloadable files

The following supplementary files are provided to demonstrate the effect of dynamically zooming on plots produced by ChromoMapper. The generated html files contain all the information to display results at different zooming levels, thus allowing to see very small objects, which do not get drawn at the standard zooming level, and to resolve regions where crowding of a large number of objects in a small space makes it impossible to see all of them independently of each other.

Supplementary File 1. Bubble plot for the *Bombus impatiens* assembly. The html file contains the plot shown in Figure 1, in the format directly produced by *ChromoMapper,* where finer details can be dynamically visualised by zooming in to expand specific areas of the plot. As in Figure 1, blocks longer than 10000 bases have been used to plot contigs (x axis) against the reference chromosome (y axis) on which they map. The alignments are represented as bubbles, whose size depends on the number of bases involved in the alignment and whose color uses a white-red-blue-black gradient to indicate highly to lowly integrated alignments. The integrity level is calculated as the ratio between maximum block and contig length.

Supplementary File 2. Alignment blocks on chromosomes for the *Bombus impatiens* assembly. The html file contains the plot shown in Figure 3A, in the format directly produced by *ChromoMapper,* where finer details can be dynamically visualised by zooming in to expand specific areas of the plot. As in Figure 3, for each chromosome reported on the y axis, alignment blocks are reported as rectangles coloured according to the contig they belong to, positioned at the reference genomic location on which they map (Mbases). Interruptions between contigous alignment blocks are represented as rectangle interruptions. Horizontal lines connect two contigous blocks from the same contig.

Supplementary File 3. Alignment gaps on chromosomes for the *Bombus impatiens* assembly. The html file contains the plot shown in Figure 3B, in the format directly produced by *ChromoMapper,* where finer details can be dynamically visualised by zooming in to expand specific areas of the plot. As in Figure 3, for each chromosome reported on the y axis, gaps are represented as black rectangles as a function of reference genomic position (Mbases).

Supplementary File 4. Contigs on human chromosome 22. The html file contains the plot shown in Supplementary Figure 3 for chromosome 22 from the GRCh38.p14 mapped on the human T2T assembly, in the format directly produced by ChromoMapper, where finer details can be dynamically visualised by zooming in to expand specific areas of the plot. As in Supplementary Figure 3, each contig is reported on the y axis and alignment blocks are reported as coloured rectangles located at the reference chromosome position (Mbases) on which they are mapped. Thinner lines are used to connect blocks of the same contig which are not contiguous on the chromosome.

References

Salzberg,S.L. *et al.* (2012) GAGE: A critical evaluation of genome assemblies and assembly algorithms. *Genome Res*, **22**, 557–567.
